# AI‐Driven Integration of Minority Stress, Gender‐Affirming Hormone Therapy, and Epigenetic Aging in Alzheimer's Disease Progression in Transgender Individuals

**DOI:** 10.1002/alz70855_102583

**Published:** 2025-12-23

**Authors:** Rifaldy Fajar, Sahnaz V Putri, Prihantini Prihantini

**Affiliations:** ^1^ Yogyakarta State University, Sleman, Special Region of Yogyakarta, Indonesia; ^2^ International University Semen Indonesia, Gresik, East Java, Indonesia; ^3^ Bandung Institute of Technology, Bandung, West Java, Indonesia

## Abstract

**Background:**

The intersection of minority stress, epigenetic aging, and Alzheimer's disease (AD) progression is a critical yet underexplored research area, particularly among transgender individuals undergoing gender‐affirming hormone therapy (GAHT). Minority stress, defined as chronic stigma and discrimination, is hypothesized to accelerate biological aging and exacerbate neurodegeneration. This study applies an AI‐driven multi‐modal approach to explore the relationships between minority stress, epigenetic aging, and AD progression.

**Method:**

This study integrated publicly available datasets to investigate the research objectives. DNA methylation data specific to Alzheimer's disease patients (*n* = 161) were sourced from the Gene Expression Omnibus (GSE153712) to identify stress‐sensitive CpG sites linked to epigenetic aging. Neuroimaging data were obtained from the Open Access Series of Imaging Studies (OASIS‐3, *n* = 598), focusing on hippocampal atrophy and cortical thinning. Socio‐demographic and clinical data from the UK Biobank (*n* = 2,230) were analyzed to capture mental health metrics, socio‐economic factors, and proxies for minority stress. Due to the lack of transgender‐specific data, GAHT exposure and minority stress variables were modeled using synthetic augmentation techniques validated with independent cohorts from the National Alzheimer's Coordinating Center (NACC, *n* = 200). Data harmonization was achieved using a Transformer‐based Variational Autoencoder, while epigenetic marker discovery and neurodegeneration modeling utilized ensemble Random Forests and Temporal Graph Convolutional Networks (T‐GCN). External validation was performed using cohorts from NACC and OASIS.

**Result:**

The T‐GCN model showed strong predictive performance (AUC: 0.86, 95% CI: 0.81–0.89; F1‐score: 78.4%). Minority stress was associated with an average epigenetic age acceleration of 6.2 years (95% CI: 5.4–7.0), with significant CpG methylation changes in FKBP5 and NR3C1 genes (*p* <0.001). Accelerated epigenetic aging correlated with reduced hippocampal volume (β=‐0.30, *p* <0.001) and increased amyloid‐β deposition (*p* = 0.004). Social support mitigated epigenetic aging by 21% (95% CI: 15–27%). Validation confirmed the consistency of these findings across cohorts.

**Conclusion:**

This study presents a novel AI‐driven framework for understanding minority stress‐induced epigenetic aging and its impact on AD progression. By using advanced computational techniques, it addresses critical gaps in inclusive AD research and contributes to precision healthcare.

